# Impact of interspecies colostrum and milk replacement on circulating sncRNA dynamics of neonatal goat kids

**DOI:** 10.1080/15476286.2026.2692293

**Published:** 2026-06-26

**Authors:** Rob J. Dekker, Marielle H. van den Esker, Lars Ravesloot, Wim C. de Leeuw, Marina van Olst, Martijs J. Jonker, Timo M. Breit, Ad P. Koets

**Affiliations:** a RNA Biology Research Group, Swammerdam Institute for Life Sciences, Faculty of Science, University of Amsterdam, Amsterdam, The Netherlands; b Department of Bacteriology, Wageningen Bioveterinary Research, Lelystad, The Netherlands

**Keywords:** Circulating RNA, small untranslated RNA, colostrum, milk substitutes, goats

## Abstract

Replacing maternal colostrum and milk with those from other species is common practice in neonatal ruminant management, but the effects on small non-coding RNA (sncRNA) transmission remain poorly understood. This study investigated whether colostrum and milk replacement alters circulating sncRNA profiles in neonatal goats using a twin-pair model. Six vaccinated goat dams (*Mycobacterium avium* ssp. *paratuberculosis*) and their twelve kids were included. One goat twin kid received maternal colostrum and milk, while the other was fed a replacement diet with bovine colostrum and milk replacer. Small RNA sequencing was performed on serum samples from kids, as well as on goat colostrum and milk and on bovine replacers. All colostrum and milk samples contained diverse sncRNAs, including miRNAs, and tRNA- and rRNA-derived fragments. Processing and storage influenced sncRNA abundance and stability. In neonatal serum, sncRNA profiles changed during development and indicated two response waves: after colostrum replacement and after three weeks of continued milk replacement. These changes involved differential abundance of miRNAs and rRNA-derived fragments, including molecules linked to immune regulation, metabolism, and developmental pathways. Although sequence similarity between bovine and caprine sncRNAs limited direct inference of dietary transfer, the observed differences suggest that milk origin and composition may influence circulating sncRNA profiles. Several milk-associated miRNAs were consistently detected in serum, suggesting potential maternal transfer. Overall, these findings indicate that interspecies milk replacement may modify neonatal circulating sncRNAs, highlighting a potential role for milk-derived sncRNAs in shaping early-life immune and developmental programming.

## SUMMARY

In dairy goat farming, newborn kids are often fed cow colostrum and milk replacer instead of their mother’s milk. This study examined how that practice affects circulating small non-coding RNAs in blood of goat kids. These molecules play a role in regulating gene activity. We found that the type and abundance of these RNAs changed, especially after three weeks of feeding milk replacer. Since small non-coding RNAs are involved in immunity, metabolism, and brain development, the observed changes may have lasting effects on the kids’ health. Our findings highlight how early-life nutrition influences biological development in important and complex ways.

## Introduction

For newborn ruminants, such as calves and goat kids, the first weeks of life are a critical period in terms of development and survival. Ruminants are born with a functional but naïve immune system and are agammaglobulinemic due to an epitheliochorial placenta, which precludes in-utero transfer of macromolecules such as immunoglobulins [1]. Therefore, colostrum and milk uptake are essential for early neonatal immunity and protection. Colostrum, the first secretion of the mammary gland following parturition, has a complex composition that varies between species, individual animals and even between mammary glands within individuals. In general, bovine colostrum contains higher concentrations of bioactive proteins, lipids, growth factors and certain vitamins compared to bovine milk [2].

The protein fraction of ruminant colostrum contains large amounts of bioactive immunoglobulins, predominantly of the IgG1 isotype, and timely colostrum intake within the first 24 hours of life is essential for passive transfer of immunity to ruminant neonates [3]. Following absorption across the gastrointestinal epithelium, antibodies reach serum, as well as other tissues of neonatal goat kids, where they provide early immunological protection [4]. Ruminant IgG antibodies exhibit a transient presence in serum, with an estimated half-life of 10–26 days in general, and specifically 11 days in goat kids [5–7]. As a result, by approximately three weeks of age, most colostrum derived IgG antibodies are no longer detectable in serum. In addition to antibodies, colostrum-derived immune cells can be transferred to neonates while retaining antigen specific functionality, supporting cellular immunity in newborns [7,8].

While the primary recipient of bovine colostrum is the dam’s own calf, the diverse array of bioactive constituents also makes it suitable for development of (interspecies) functional food with veterinary as well as human health applications [9]. In the veterinary field, bovine colostrum is successfully used to raise dairy goat kids in programmes aimed at preventing and eradicating caseous lymphadenitis (CL), caprine arthritis encephalitis (CAE) and *Mycobacterium avium* ssp. *paratuberculosis* (MAP). Such programmes prevent the transmission of infection from the goat dam to their kids while securing transfer of passive immunity [4,10,11].

Although antibodies are the most well-studied components of colostrum and milk, the practical use of bovine colostrum and milk in interspecies rearing raises questions about other constituents, whose uptake, dynamics and functional impacts are less well understood [12]. These constituents include small non-coding RNAs (sncRNAs) that are abundantly present in colostrum and milk, often in the context of extracellular vesicles [13–15]. Small non-coding RNAs are short RNA molecules that regulate cellular processes and gene-expression, for instance by sequence-specific posttranscriptional gene regulation [16]. Human studies have shown that sncRNAs are transferred from the mammary gland to milk [17] and subsequently to infants, thereby directly influencing regulatory pathways, including pathways involved in immunology, neurogenesis and glucose metabolism [18–20]. Hence, these sncRNAs may play an important role in neonatal development.

The high concentrations of sncRNAs in ruminant colostrum and milk suggest that these molecules have a biological role as well. The sncRNA profile of colostrum appears to be influenced by nutrition and environmental factors [21], suggesting that the maternal environment influences milk-mediated transfer of sncRNAs. Since substitution of goat colostrum by bovine colostrum to raise goat kids is an interspecies (xenogeneic) replacement, sncRNA transfer may be altered. However, the dynamics, functional implications, and broader nutrigenomic aspects of interspecies sncRNA transfer remain largely unknown [22].

Therefore, the current study aimed to investigate the effect of interspecies colostrum and milk replacement on qualitative and quantitative aspects of circulating sncRNAs in goat kid serum by identifying which sncRNAs are affected and exploring their potential biological functions. A goat twin model was employed, in which one twin-kid received maternal colostrum and milk while the other twin-kid was fed bovine colostrum and milk replacers. This design enabled comparison of sncRNA profiles in colostrum, milk, and serum between the two genetically similar kids from the same dam. Small non-coding RNA profiles are often species-specific, influenced by maternal environmental factors, sensitive to milk treatment and storage conditions and involved in regulating diverse cellular and developmental processes. For this reason, xenogeneic colostrum and milk feeding is expected to modulate circulating sncRNA profiles.

## Materials and methods

### Experiment

All aspects of the goat twin model experiment have been described in detail in a publication of a study on transfer of maternal antigen-specific cellular immunity against MAP via colostrum (Experiment 1 in [7]). The current study was conducted on biobanked material of this study as described in the supplementary materials and methods, and only aspects essential to comprehend the current work will be repeated here. This research was conducted in accordance with the ARRIVE guidelines. The study was reviewed and approved by the Utrecht University Animal Ethics Committee and the Dutch Central Committee for Animal Experiments under permit number AVD1080020185064. Animal experiments were conducted in accordance with the Dutch law on Animal Experimentations (Wet op de Dierproeven) and European regulations on the protection of animals used for scientific purposes (EU directive 2010/63/EU).

The original experiment included 12 goat dams and their 24 kids, of which 6 kids were chosen from the kids in the GC group (see below) using the =RAND() function in Excel to assign a random number to the 12 and picking the lowest 6 numbers. Subsequently the 6 matching siblings and the 6 twins dams were identified and selected this sncRNA experiment. Hence, six biological replicates were included in each group to ensure adequate statistical power while accounting for biological variability [23]. The dams were immunized with an inactivated MAP vaccine approximately 2 weeks prior to the mating season. The vaccine-induced immune response was evaluated after 100 days with tuberculin (PPD-A) skin testing. Only twin-kids from dams showing a positive skin test response during gestation were included in the experiment. The dams were monitored 24/7 on CCTV during the last three weeks of gestation. Birthing started naturally at a gestation length of approximately 145 days after natural breeding. Birthing was fully supervised, and kids were removed from the dam and monitored directly after birth to prevent spontaneous suckling by newborns.

**Figure 1.**
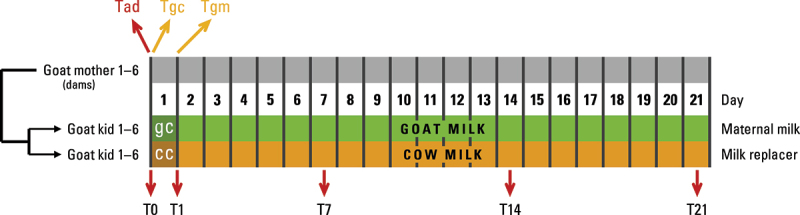
Schematic overview of the twin-goat experimental setup designed to assess the influence of early nutrition on serum small non-coding RNA (sncRNA) presence. Newborn twin goats were assigned to either maternal colostrum/milk (control: GC) or cow colostrum/milk replacer feeding groups (CC). Serum samples were collected from each dam (Tad) and kid at the indicated time points (red arrows). From each dam, one maternal colostrum sample (Tgc) and one milk (Tgm) sample were also collected (yellow arrows). Additionally, three cow colostrum (cc) samples obtained immediately post-calving (pasteurized and frozen/thawed), and one commercial milk replacer sample were included. In total, 82 biological samples were analysed.

The newborn twins were allotted over two treatment regimens using a predefined pseudo-random block randomization scheme to ensure that 1st/2nd born kids and male/female kids were equally distributed between the two treatments. Twin pairs of goat kids born from dams were either fed maternal colostrum and milk from their dam (kid 1) or pasteurized and frozen/thawed bovine colostrum from certified MAP free, non-vaccinated cows followed by commercial bovine milk replacer (Denkamilk Capriplus, 22% crude protein; Denkavit Nederland BV, Voorthuizen, The Netherlands) (kid 2). All kids were either bottle-fed fresh maternal goat colostrum (GC) within 90 minutes after birth, or thawed cow colostrum (CC) (warmed to 39°C directly prior to feeding). Both kids received a volume of colostrum based on 5% of bodyweight at birth in ml. Subsequently, kids were individually bottle-fed twice daily with milk from their own dam directly after milking (GC) or milk-replacer (CC) according to industry standard protocols and instructions that came with the milk-replacer product. The IgG concentration in colostrum of the goat dams (*n* = 6) was 32.7 mg/ml (±14.1, 1 SD) in the first milking after parturition, and 31.7 mg/ml (±12.5, 1 SD) in the second milking. In comparison, the colostrum of the cow contained 114.4 mg/ml IgG. Despite differences in colostrum IgG concentrations, the mean IgG uptake was comparable over time and across groups (Supplementary Figure SF1). The newborn twins thus represent two treatment regimens: Group 1 (Goat colostrum, GC) received colostrum and milk derived from their dam; Group 2 (Cow colostrum, CC) received pasteurized and frozen/thawed bovine colostrum in similar quantities as kids of Group 1. A schematic overview of the experiment is shown in Figure 1.

### Sample collection

During the experiment (Figure 1, Supplementary Table ST1), jugular blood samples (serum vacutainer tube) were collected from all goat kids prior to colostrum feeding (T0) as well as on days 1, 7, 14, 21. Immediately following parturition (T0), blood samples were also collected from all dams. At 2–4 hours (T0) and ~24 hours (T1) postpartum, dams were machine-milked to obtain maternal colostrum. Also, 20 ml samples were collected from three pasteurized plus frozen/thawed bovine colostrum pools used as maternal milk replacement and Denkamilk Capriplus milk replacer (28.5 g dissolved in 150 mL water).

### Small RNA isolation

The miRNeasy Serum/Plasma kit (Qiagen) was used to isolate and purify RNA from 200 μl fresh frozen serum or milk. Isolated RNA was examined using the 2200 TapeStation System with Agilent High Sensitivity RNA ScreenTapes (Agilent Technologies).

### Small RNA-seq

Small RNA-Seq libraries were generated from RNA according to manufacturers’ protocols using the Small RNA-Seq Library Prep Kit (Lexogen). The size distribution of libraries with indexed adapters was assessed using a 2200 TapeStation System with Agilent D1000 ScreenTapes (Agilent Technologies). Libraries were quantified on a QuantStudio 3 Real-Time PCR System (Thermo Fisher Scientific) using the NEBNext Library Quant Kit for Illumina (New England BioLabs) according to instructions of the manufacturer. Libraries were clustered and sequenced (75 bp) on a NextSeq 550 Sequencing System (Illumina) using a NextSeq 500/550 High Output Kit v2.5 (75 Cycles) (Illumina).

### Read quality control and preprocessing

Initial quality control of raw sequencing data was performed using MultiQC v1.21 [24] and FastQC v0.11.9 [25]. Two samples were excluded from further analysis based on deviating quality metrics and outlier positions in principal component analysis (PCA), as shown in Supplementary Table ST2 and Supplementary Figure SF2. These samples belonged to the maternal-fed group (T0) and the other to the cow-replacer fed group (T1), leaving five instead of six replicates at these time points. Read preprocessing was carried out in multiple stages: low-quality bases and adapter contamination were removed using Trimmomatic v0.39 [26] with the parameters LEADING:3 TRAILING:3 SLIDINGWINDOW:4:15 ILLUMINACLIP:adapters.fa:3:10:4. Cutadapt v4.6 [27] was used to remove reads containing 5′ adapter sequences. Additionally, reads mapping to the Illumina control genome Escherichia coli phage phiX174 (NC_001422.1) were removed using Bowtie2 v2.4.1 [28] with default settings.

### Read filtering, mapping, and normalization

After pre-processing, two separate count tables were generated: one for serum samples (minimum 132 reads per unique sequence) and one for milk samples (minimum 32 reads). Batch correction was applied using the *ComBat_seq* function from the sva package v3.50.0 [29] in R v4.3.2 [30] to adjust for RNA isolation blocks 1 and 2; blocks 3 and 4 were left uncorrected (Supplementary Table ST1). Unique sequences from both serum and milk datasets were mapped using Bowtie2 (default settings) to 20 distinct ncRNA classes, each comprising all known reference sequences belonging to that class, as detailed in Supplementary Table ST3. For each sncRNA class, two count tables were generated: one with unique sequences (USs) as rows and samples as columns, showing read counts per unique sequence per sample (Supplementary Table ST4); and a second with class members as rows, where each cell contains the aggregated reads of all unique sequences mapping to that member (Supplementary Table ST5). Although 76-nt reads mapping to lncRNAs may represent larger sncRNAs, their low abundance, combined with size-selection steps inherent to the sRNA-seq protocol, ensures that the vast majority of sRNA-seq reads represent genuine sncRNA molecules.

### Differential expression and species-specific analysis

Differential expression analysis, including normalization, was performed using a linear mixed model approach described by Hoffman and Roussos [31], implemented via the *voomWithDreamWeights* function from the variancePartition package v1.32.5 [32] in R, producing fold-changes and adjusted *p*-values. Adjusted *p*-values below 0.05 were considered statistically significant. Differential expression analyses were performed for time-series within both groups, comparing T1, T7 and T21 to T0 for both the GC and CC groups separately. In addition, a direct comparison was made between both groups, where all time points of the GC and CC group were compared (T0 of GC versus T0 of CC, T1 of GC versus T1 of CC, etc.).

To identify bovine-specific non-coding RNAs, sequences were mapped to both goat (GCF_001704415.1) and cow (GCF_002263795.2) reference genomes using Bowtie2 [28]. Unique sequences that poorly mapped to the goat genome but showed high-quality alignment to the bovine genome were selected based on mapping quality (see Supplementary Table ST6). Finally, the secondary structure of 18S rRNA was predicted using RNAfold [33] to assess thermodynamically stable conformations.

### Target prediction of miRNAs and functional analysis of target genes

The mRNA targets of differentially expressed miRNAs were either accessed via miRbase [34] or predicted using computational target prediction algorithms TargetScan v7.0 [35] and miRanda v3.3a [36]. 3’ UTR regions were extracted from the *Capra hircus* genome GCF_001704415.1, and miRNAs were selected from differentially expressed miRNAs with log2foldchanges < −1 or >1. MiRanda and TargetScan were run using default settings, 6-mers were removed from TargetScan hits. The intersect of both programmes were considered as putative mRNA targets. The biological function of these mRNA targets was further analysed using clusterProfiler in R [37]. Biological pathways were annotated with overrepresentation analysis using the Kyoto Encyclopaedia of Genes and Genomes (KEGG) pathway [38] and gene ontology [39] and visualized using the ggplot2 package [40].

## Results

### Experimental parameters

The average weight of goat kids at birth was 3.5 kg ±0.2 kg, and the average weight at day 21 was 8.2 ± 0.3 kg. There were no significant differences in weight or growth between treatment groups. On average, male goat kids tended to be slightly heavier at birth and at 7, 14 and 21 days of age compared to female kids, however these differences were not statistically significant.

After sample collection and sncRNA isolation from all samples, subsequent sRNA-seq yielded on average 6.8 ± 1.7 M raw reads per sample (Supplementary Table ST2). Sequencing data were subjected to quality control, and two samples (S9 and S22) were removed from the experiment due to insufficient data quality (Supplementary Figure SF2). After the quality control and preprocessing, 4.5 ± 1.6 M reads were left for mapping.

### Most abundant sncRNA fragments in goat serum

**Figure 2.**
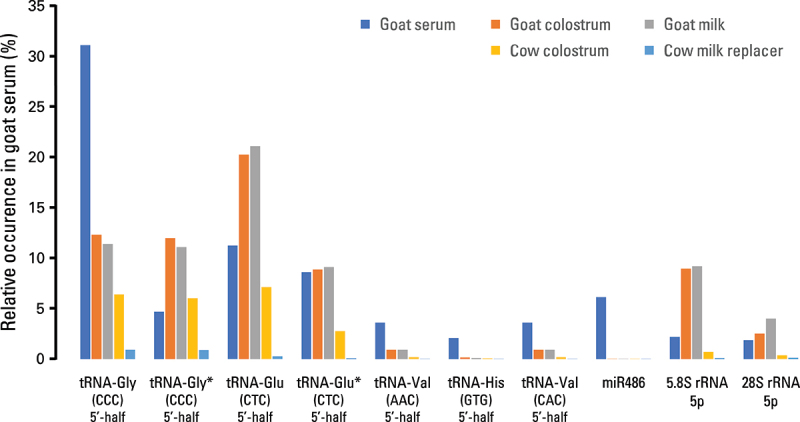
Relative abundance of the top 10 RNAs detected in goat serum. Bar plot showing the relative abundance of the top 10 RNA species identified in goat serum, compared across goat and cow colostrum, and milk samples (see Supplementary table ST2 for details). An asterisk (*) denotes RNA sequences that differ by a single nucleotide from the most abundant forms of tRNA-Gly-CCC or tRNA-Glu-CTC.

The ten most abundant sequenced sncRNAs in goat serum are 5’ halves from five tRNAs (Gly-CCC, Glu-CTC, Val-AAC, Val-CAC, and His-GTG), one miRNA (miRNA 486–5p), and 5’ ends of 5.8S and 28S rRNA (Figure 2, Supplementary Table ST7). These ten sncRNAs are represented by 28 sncRNA USs which make up close to 60% of the total sequenceable sncRNA molecules in goat serum. This suggests a relative limited complexity of the sncRNA serum transcriptome. This is underlined by observations that two tRNA Gly-CCC and Glu-CTC 5’ halves, each with two variants at their 5’ end, make up about 56% of all sequenced reads. Also miR-486-5p reads constitute almost 75% of all sequenced miRNA reads (Figure 2, Supplementary Table ST7).

Although no conclusions can be drawn about the absolute presence of sncRNAs in serum, colostrum or milk, the distribution of the sequenced top sncRNAs in goat colostrum and milk is generally similar, and somewhat comparable to serum sncRNAs, with the exceptions of the aforementioned miR-486 and a four times higher abundance of 5.8S rRNA-5p in goat colostrum and milk. Compared to serum, in which over one third of the sequenced sncRNAs are tRNA-Gly-CCC 5’ halves, goat colostrum and goat milk appeared to contain a considerable amount of 5.8S rRNA-5p. This is not the case for the cow colostrum and cow milk replacer used in this experiment (Figure 2, Supplementary Table ST7). In contrast, in the group fed cow milk, the most abundant sncRNAs detected in serum by sequencing represent only 2.4% of the sRNA-seq reads.

### Differential sncRNA presence in goat serum during early development

To get an impression of the dynamics of sncRNAs in serum during early goat development, differential presence of sncRNAs (DiPRs) was determined for goat serum time series of kids that received either normal maternal milk or milk replacer. Comparing all time points in one group to the associated T0 revealed many sncRNAs that showed differential presence during early development (Table 1, Supplementary Table ST8). These DiPRs were observed across all types of ncRNA, with lncRNAs and tRNA-derived fragments showing the lowest levels of differential presence. There appears to be an increase in DiPRs over time, although there may be a slight increase at T1. All these DiPRs found during early goat development indicate that relative presence of individual sncRNAs in serum can vary substantially. This is also exemplified by a clear distinction in distribution of samples in PCA of miRNA sRNA-seq reads (Supplementary Figure SF3B). Virtually all RNA types showed considerably more DiPRs during development in serum from kids fed milk replacer compared to those fed maternal milk.

**Table 1.** Serum non-coding RNAs differentially present during early goat development.

		Differential unique sequences (USs) in serum*											
		Kids fed with maternal milk						Goats fed with milk replacer					
RNA Type	#	T1	T7	T14	T21	All	%	T1	T7	T14	T21	All	%
lncRNA	726	0	3	8	8	12	2	26	24	17	21	55	8
miRNA	335	16	12	45	33	71	21	36	12	20	49	87	26
rRNA	122	2	1	5	34	36	30	49	30	1	52	67	55
rRNA parts	26	0	2	0	7	8	31	12	6	0	9	14	54
scRNA-parts	5	3	2	2	1	3	60	2	2	0	2	4	80
snRNA	110	0	3	4	5	11	10	0	13	3	15	23	21
snRNA-parts	106	4	3	7	1	11	10	1	10	0	9	17	16
snoRNA	97	4	8	4	9	14	14	15	9	2	26	32	33
snoRNA-parts	95	6	9	4	9	15	16	17	13	1	27	35	37
SRP-RNA-parts	23	0	0	1	1	2	9	1	2	0	2	4	17
tRNA	52	0	1	0	2	3	6	8	2	1	19	22	42
vault-RNA-parts	2	2	0	0	0	2	100	1	0	0	0	1	50
Y-RNA-parts	13	1	0	1	2	2	15	2	1	0	6	7	54
Total	1,712	38	44	81	112	190	11	170	124	45	237	368	21
Other RNA (US)	26,076	44	15	139	157	287	1	413	240	96	1,080	1,475	6

Notes: *, numbers indicate unique RNA sequences (USs) that differed in serum abundance at each postnatal timepoint compared with T0 in kids fed maternal milk or milk replacer; T0, before first feeding; T1, 1 day after birth; T7, 7 days after birth; T14, 14 days after birth; T21, 21 days after birth. US, unique sequence. #, total number of detected unique sequences for each RNA type. All, number of unique sequences differentially present at one or more timepoints. Because the same US can be differential at more than one time point, ‘All’ may be smaller than the sum of the time point columns. %, ‘All’ as a percentage of ‘#’. RNAs were considered differentially present when the adjusted *p* value was < 0.05.

**Figure 3.**
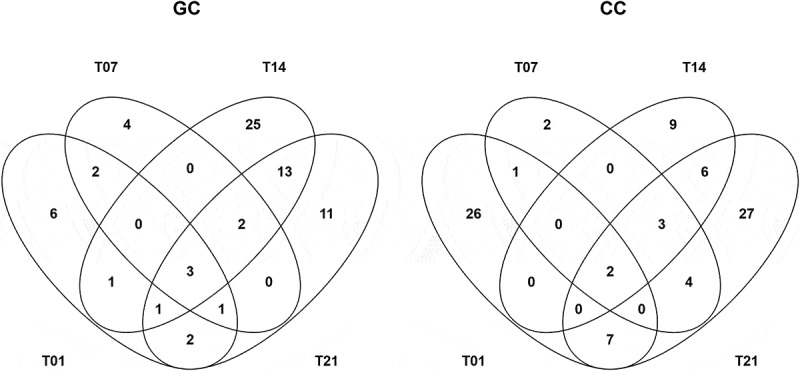
Venn diagrams of miRNAs that are differentially present in goat kid serum at T1, T7, T14 and T21 when compared to T0. In serum of goat kids in the group fed maternal goat milk (GC), three miRNAs were differentially present at all time points (miR-29a-3p, miR-2889 and miR-6923-3p), while in the milk replacer group (CC) two miRNAs were differentially present in serum of goat kids (miR-6027-5p and miR-6651-5p).

Among all sncRNAs, the function of miRNAs is best understood, and their function can either be accessed via databases, or predicted using specific tools that identify mRNA targets. The biological function of miRNAs that were differentially present at all time points (T1, T7, T14 and T21) was further investigated. When goat kids are fed maternal milk, three miRNAs are differentially present at all time points: miR-29a-3p, miR-2889 and miR-6923-3p (Figure 3). Interestingly, these miRNAs are also present in goat milk (Supplementary Table ST9). These three DiP miRNAs in the goat milk group are not identified as DiPRs in the cow milk-fed group, except at T1. In the cow group, two miRNAs are differentially present at all time points: chi-miR-6027-5p and chi-miR-6651-5p. Milk replacement therefore appears to be associated with a different profile of serum miRNAs in goat kids compared with maternal feeding.

### Bovine-specific sncRNA presence in goat serum after milk replacement

To identify bovine-specific USs potentially transferred from cow colostrum and cow milk to goat kid serum, all USs were mapped against both the caprine and bovine genomes. All USs that mapped poorly to the caprine genome but sufficiently to the bovine genome, were selected (Supplementary Table ST6). This approach identified 63 bovine-specific USs, corresponding to four sncRNA: bta-miR-2285t, snoRD29, vault-RNA3-1, and Y3 RNA. Most of these reads originated from a single sample (S44, serum of kid on bovine milk replacement from dam 92,963 at T14, Supplementary Table S6) and read counts were extremely low. Altogether, the number of reads uniquely mapping to bovine-derived sncRNAs in goat kid serum was very low due to high sequence homology with caprine sncRNAs, preventing determination of their origin.

### Serum sncRNA changes reflect milk replacer composition

**Figure 4.**
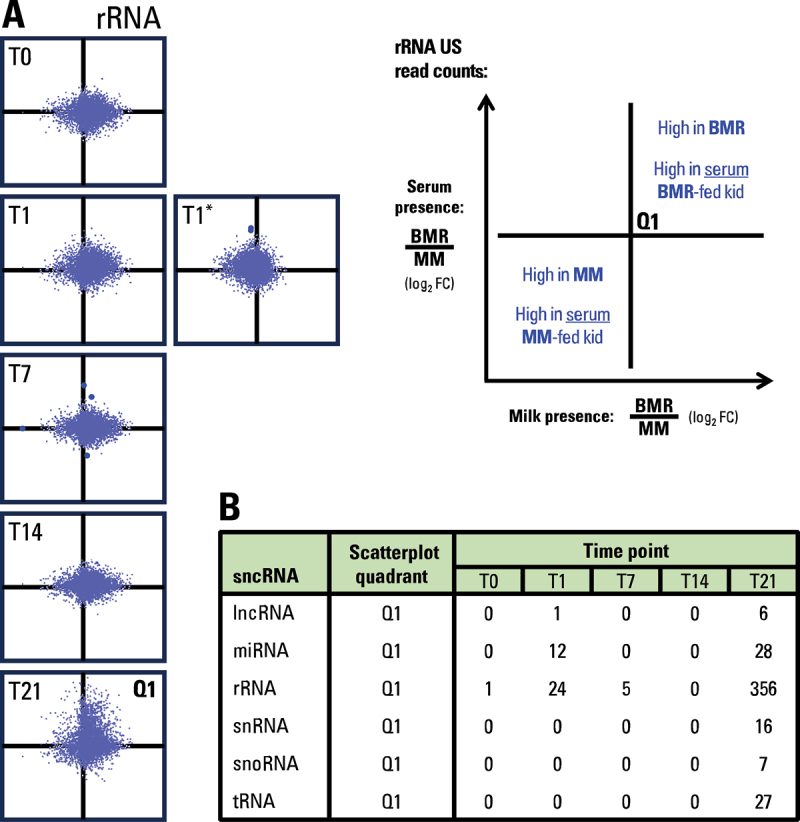
Relationship between milk and serum presence of sncRNAs. (A) Scatterplots illustrating the relationship between rRNA presence in milk and serum. Each point represents the log_2_ Fold change in read counts for a given rRNA-derived unique sequence (US), comparing bovine milk replacer (BMR) to maternal milk (MM; Tgm in Figure 1) on the x-axis, and serum from kids fed BMR versus serum from twin kids fed MM on the y-axis. The T1* panel specifically compares the log_2_ Fold change between bovine colostrum and goat colostrum (Tgc in Figure 1). A schematic scatterplot is provided to aid interpretation, with quadrants reflecting concordant or discordant sncRNA abundance between milk and serum. (B) Table summarizing, for each sncRNA class, the number of USs with differential presence at T21. Shown are USs with a Fold change ≥ 2 in BMR vs. MM and with statistically significant differences (adjusted *p*-value ≤ 0.05) in serum between BMR-fed and MM-fed kids. Q1 indicates the quadrant highlighted in the scatterplots of panel A. Scatterplots for all analyzed sncRNAs are provided in Supplementary Figure SF3.NA.

Due to the high sequence homology between bovine and goat sncRNAs, bovine-specific sncRNAs could not be reliably annotated. Therefore, the effect of milk replacement on serum sncRNA presence was assessed by comparing sncRNA expression between maternal-milk-fed (GC) and bovine-milk-fed (CC) kids at different time points. This design provided an opportunity to determine whether milk replacement may be associated with altered serum sncRNA profiles. The ratio of milk sncRNAs presence in caprine maternal milk versus bovine milk replacer was plotted against the ratio of serum sncRNA presence in maternal-milk-fed and bovine-milk-fed kids (Figure 4(A), Supplementary Figure SF3). Subsequently, sncRNAs with differential presence (DiP) in either contrast were summarized (Figure 4(B), Supplementary Table ST10). A relation between DiPRs in bovine milk replacer compared to maternal milk and DiPRs in the serum of bovine-milk-fed versus maternal-milk-fed kids was observed. This effect was most obvious at T21, with a slight transient effect at T1. Quadrant 1 contained the highest number of DiPRs, especially for rRNA fragments (Figure 4(A), Supplementary Table ST10).

### Differential sncRNA presence in goat serum after milk replacement

**Figure 5.**
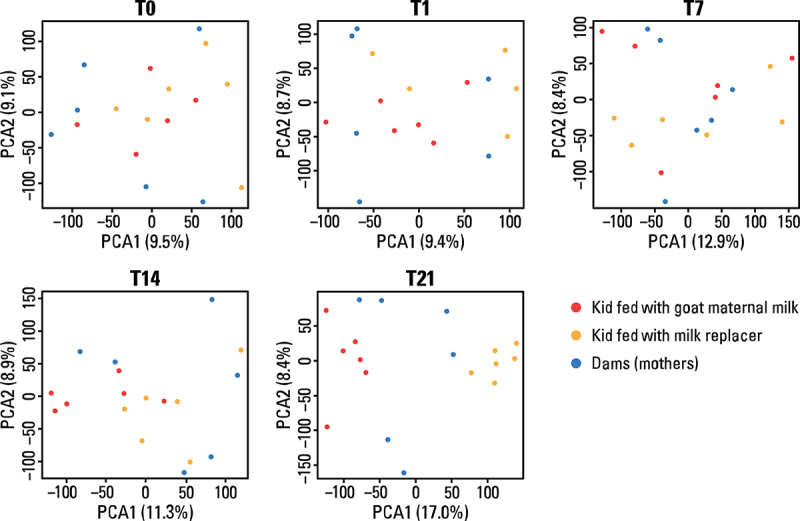
Principal component analysis (PCA) of serum sncRNA profiles across developmental time points. PCA plots showing the distribution of serum small non-coding RNA (sncRNA) expression profiles at five time points (T0, T1, T7, T14, and T21). Data represent three experimental groups: kids fed with goat maternal milk (red dots), kids fed with milk replacer (orange dots), and dams (blue dots). Each plot illustrates the first two principal components (PCA1 and PCA2), with the percentage of variance explained indicated on each axis. Separation of groups over time highlights dynamic changes in serum sncRNA composition associated with diet and developmental stage.

To assess differential presence of serum sncRNAs after milk replacement, a PCA analysis was performed comparing, at each time point, serum USs from goat kids, as well as their adult mothers. The PCAs of all sRNA-seq reads (Figure 5) indicate an increasing separation over time between serum scnRNA profiles of maternal-milk-fed kids and milk-replacer-fed kids. At T21, PCAs point to complete separation of serum sncRNA profiles between kids fed with milk replacer and those fed with goat maternal milk.

To further analyse the impact of milk replacement on serum sncRNA (fragments), differential presence was determined for serum USs and sncRNAs (Table 2 and Supplementary Tables ST8 and ST9). All types of sncRNA appeared to be differentially present both as USs and as sncRNAs. Most DiPRs were detected at T21, although an early peak at T1 was observed, most prominently for miRNAs (Table 2). Comparison of these results with the DiPRs showed similar numbers for sncRNA, except for snoRNA, tRNA (up), and miRNA (down). Differential serum presence of tRNA-derived fragments was observed. While little change in the presence of tRNA-derived fragments was observed over development time, 42% of all possible tRNA-derived fragments showed altered presence following milk replacement (Table 2).

**Table 2.** Unique sequences and annotated non-coding RNAs in serum showing differential presence in milk-replacer-fed goat kids compared with maternal-milk-fed goat kids.

		Differential unique sequences (USs) in serum*						
RNA Type	#	T0	T1	T7	T14	T21	All	%
lncRNA	1,286	0	7	0	0	19	26	2
miRNA	2,747	0	59	0	0	151	204	7
rRNA	11,785	9	92	10	2	1,515	1,589	13
rRNA parts	3,309	0	52	16	13	924	969	29
scRNA-parts	18	0	0	3	2	7	10	56
snRNA	492	0	6	0	0	58	64	13
snRNA-parts	2,880	0	5	0	0	51	56	2
snoRNA	230	0	8	2	2	43	50	22
snoRNA-parts	209	0	9	1	2	41	50	24
SRP-RNA-parts	256	3	0	0	0	24	26	10
tRNA	3,686	0	3	0	0	148	150	4
vault-RNA-parts	20	0	0	0	0	2	2	10
Y-RNA-parts	756	1	1	0	0	54	56	7
Other RNA (US)	26,076	3	11	2	12	1,043	1,065	4
Total	53,750	16	253	34	33	4,080	4,317	8
		Differential sncRNAs in serum*						
lncRNA	726	0	3	0	0	4	7	1
miRNA	335	0	23	0	1	17	39	12
rRNA	122	0	3	0	0	41	41	34
rRNA parts	26	0	4	0	1	10	13	50
scRNA-parts	5	0	1	0	1	2	3	60
snRNA	110	0	0	0	0	11	11	10
snRNA-parts	106	1	0	0	0	10	11	10
snoRNA	97	0	1	1	0	26	26	27
snoRNA-parts	95	0	9	1	2	41	50	53
SRP-RNA-parts	23	0	0	1	0	4	4	17
tRNA	52	2	0	0	0	21	22	42
vault-RNA-parts	2	0	2	0	0	0	2	100
Y-RNA-parts	13	0	0	0	0	3	3	23
Total	1,712	3	46	3	5	190	232	14

Notes: *, numbers indicate unique RNA sequences (USs) that differed in serum abundance at each postnatal timepoint between kids fed milk replacer versus maternal milk; T0, before first feeding; T1, 1 day after birth; T7, 7 days after birth; T14, 14 days after birth; T21, 21 days after birth. US, unique sequence. #, total number of detected unique sequences for each RNA type. All, number of unique sequences differentially present at one or more timepoints. Because the same US can be differential at more than one time point, ‘All’ may be smaller than the sum of the time point columns. %, ‘All’ as a percentage of ‘#’. RNAs were considered differentially present when the adjusted *p* value was < 0.05.

### Differential miRNA presence

Not only are miRNAs the only RNA type with most differential expression at T1, they are also the sole RNA type with substantial DiPRs for which the number of DiPRs at T1 (*n* = 23) is bigger compared to the number at T21 (*n* = 17) (Table 2). Moreover, further analysis revealed that of 39 miRNAs with differential serum presence after milk replacement, 14 were also differentially present during normal development (Tables 2 and 3). Except for one miRNA with differential presence at T14, all others occurred at T1 and T21, with only two miRNAs overlapping between these time points. Hence, differential presence of miRNA instigated by colostrum replacement appears to be almost completely different than that caused by milk replacement. This is exemplified by one of the overlapping miRNA, miR-2889 with a higher presence at T1 and a lower presence at T21 (Table 3).

**Table 3.** Serum miRNAs showing differential presence in milk-replacer-fed goat kids compared with maternal-milk-fed goat kids.

		Fold change (log_2_)					Adjusted P value				
miRNA	DPD*	T0	T1	T7	T14	T21	T0	T1	T7	T14	T21
miR-215-3p		−0.3	**2.1**	0.9	−0.4	**3.9**	0.92	**0.03**	0.75	0.95	**0.02**
miR-2889	+	1.7	**2.1**	0.1	−1.0	**−1.8**	0.74	**0.01**	0.96	0.58	**0.02**
miR-194-5p		−0.9	1.9	0.4	1.7	1.4	0.89	0.05	0.90	0.58	0.41
miR-4492	+	0.5	**1.5**	0.7	0.2	−0.9	0.84	**0.02**	0.60	0.88	0.23
miR-26b-5p		0.5	**1.4**	0.5	0.2	−0.1	0.84	**0.01**	0.65	0.89	0.84
miR-26a-5p		0.4	**1.4**	0.4	0.4	0.0	0.85	**0.02**	0.67	0.75	0.97
miR-150-5p		0.4	**1.3**	0.4	0.1	0.2	0.88	**0.04**	0.67	0.98	0.81
miR-130b-5p		1.0	**1.3**	0.3	0.3	0.1	0.74	**0.02**	0.71	0.75	0.89
miR-155-5p		0.3	**1.3**	0.0	0.2	0.3	0.85	**0.02**	0.97	0.80	0.65
let-7e-5p		0.1	**1.2**	0.4	0.2	−0.5	0.92	**0.02**	0.67	0.88	0.52
miR-30c-5p		0.5	**1.2**	0.3	0.2	0.1	0.74	**0.02**	0.69	0.80	0.84
let-7f-5p		0.4	**1.1**	0.3	0.2	−0.1	0.74	**0.00**	0.65	0.82	0.82
miR-30b-5p		−0.1	**1.0**	0.1	0.4	0.0	0.92	**0.02**	0.86	0.64	0.94
miR-126-3p		0.1	**1.0**	0.1	0.2	−0.2	0.90	**0.03**	0.92	0.80	0.74
let-7a-5p		0.3	**0.9**	0.2	0.3	−0.1	0.75	**0.01**	0.71	0.69	0.88
miR-6529-5p		−0.3	**−0.8**	−0.2	−0.4	−0.6	0.84	**0.03**	0.67	0.58	0.08
miR-423-3p	+	−0.1	−0.9	0.1	0.1	0.4	0.93	0.05	0.92	0.98	0.48
miR-375-3p	+	−0.5	**−1.5**	−0.5	−0.8	0.2	0.84	**0.02**	0.65	0.60	0.84
miR-140-3p	+	−0.3	**−1.7**	−0.9	−0.6	0.0	0.85	**0.00**	0.30	0.63	0.97
miR-210-3p		0.2	**−1.9**	−1.4	−0.6	−0.6	0.91	**0.02**	0.23	0.69	0.51
miR-125a-3p		0.2	**−1.9**	0.4	0.1	−0.4	0.92	**0.02**	0.69	0.98	0.70
miR-193a-3p		−1.2	**−2.1**	−0.9	−0.2	0.7	0.74	**0.04**	0.62	0.92	0.56
miR-224-5p		−1.3	**−3.0**	−0.4	−1.1	−1.3	0.82	**0.02**	0.89	0.64	0.40
miR-483-3p		−1.9	−1.6	−1.1	−2.9	−2.1	0.74	0.20	0.64	0.05	0.08
miR-26b-3p	+	0.3	−1.3	4.2	1.9	**5.6**	0.92	0.66	0.07	0.64	**0.00**
miR-122-5p	+	−0.6	−0.9	1.4	2.1	**2.7**	0.84	0.44	0.37	0.06	**0.00**
miR-6239		−1.7	−0.4	1.5	0.7	**2.6**	0.74	0.84	0.42	0.80	**0.01**
miR-2285ad	+	−0.7	0.5	0.1	0.0	**2.3**	0.85	0.72	0.95	1.00	**0.03**
miR-106b-3p	+	−0.3	0.3	0.0	1.0	**1.5**	0.89	0.71	0.97	0.17	**0.00**
miR-215-5p		−0.4	0.9	0.8	0.6	**1.4**	0.89	0.20	0.63	0.67	**0.04**
miR-339-5p	+	−0.1	0.5	−0.1	0.6	**1.3**	0.95	0.41	0.95	0.63	**0.01**
miR-2340	+	−0.4	0.8	1.0	1.0	**1.2**	0.85	0.24	0.34	0.36	**0.04**
miR-451-5p	+	−0.7	0.4	−0.3	0.7	**1.2**	0.74	0.59	0.71	0.58	**0.03**
miR-11987	+	0.5	0.1	0.4	−0.2	**−1.2**	0.84	0.91	0.64	0.83	**0.02**
miR-193b-5p		−0.2	−0.9	−0.1	−0.8	**−1.7**	0.91	0.21	0.92	0.60	**0.02**
miR-12034		−0.5	0.8	0.6	0.6	**−1.7**	0.84	0.26	0.64	0.66	**0.02**
miR-5126		0.8	1.3	1.2	−0.2	**−2.2**	0.86	0.15	0.62	0.93	**0.02**
miR-493-5p		−0.9	−0.2	−0.8	−1.3	**−2.4**	0.84	0.85	0.65	0.58	**0.04**
miR-136-3p		−0.2	−0.8	−1.5	−1.1	**−2.8**	0.92	0.51	0.60	0.64	**0.02**

Notes: *, miRNA species with differential presence during development (DPD; see Table 1) are marked ‘+’; Bold text, miRNAs that are differential at a given timepoint with *p* < 0.05.

Given the relatively small number of DiPRs, it is noticeable that three sets of miRNA occur; miR-215-5p/3p, miR-26a-5p/b-3p/5p, and miR-193a-3p/b-5p, either at T1 or at T21. In addition, miR-26b was among the most abundantly sequenced miRNAs in milk.

**Figure 6.**
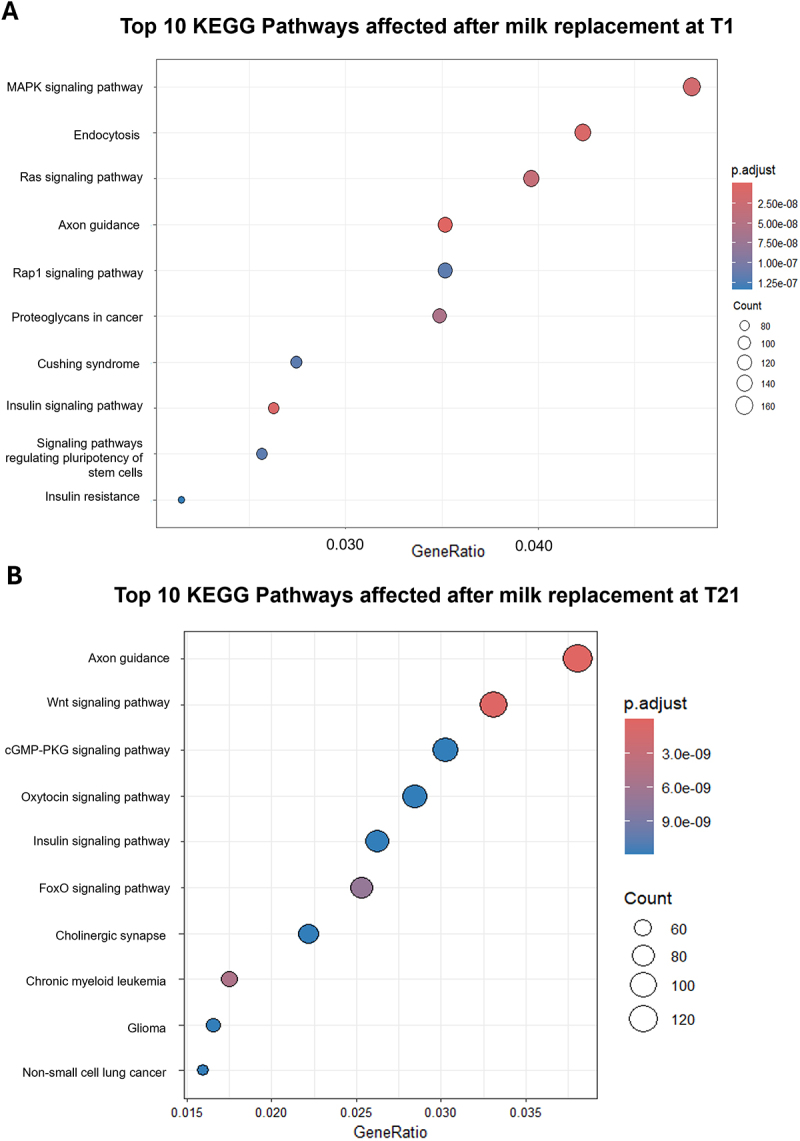
KEGG analysis of DiPRs between maternally fed and cow-fed goat kids at T01 and T21. In A, the top 10 significantly KEGG pathways identified by over-representation analysis from DiPRs are shown at time point 1. In B, the top 10 KEGG pathways identified by over-representation analysis from DiPRs are shown at time point 21.

The mRNA targets of the miRNAs that were differentially present at T1 and T21 and had a log2-fold change higher than 1 or lower than −1 were predicted using the intersect of miRanda and TargetScan algorithms. In Figure 6, predicted KEGG biological pathways that are influenced by milk replacement at both time points are shown. Although overlap between miRNAs that are differentially present at both time points is relatively small, predicted enriched pathways that are influenced by these miRNAs do partly overlap. This overlap corresponds to the presence of −5p and −3p forms. Not only miRNA -a and -b are present at both time points (e.g. miR-215-5p and −3p), but also other miRNAs might target mRNAs that act in similar pathways.

At time point 1, metabolic pathways related to glucose metabolism appeared to be strongly affected by colostrum replacement: the top 10 enriched KEGG pathways include Cushing’s syndrome, insulin signalling pathway and insulin resistance (Figure 6(A)). At time point 21 this effect is still observed (insulin signalling pathway), indicating continued effects on glucose-related pathways after milk and colostrum replacement (Figure 6(B)). Another process affected at both time points is neurogenesis and neuronal maturation. The KEGG pathway analysis suggested significant enrichment of the axon guidance pathway at T1 and T21. This is supported by GO terms analysis, where at T1 glutamatergic synapse, dendrite and postsynaptic density membrane were enriched, while at T21, enriched GO terms included glutamergic synapse, synapse, dendrite, axon, postsynaptic density and postsynapse (Supplementary Figure SF4). Other enriched pathways were related to signal transduction, (stem) cell differentiation and proliferation, including endocytosis, MAPK signalling, Ras signalling, Rap1 signalling, Wnt signalling and pathways involved in regulating pluripotency of stem cells (Figure 6). Overall, the enriched pathways at both time points involve cell development, glucose metabolism and nervous system differentiation and development.

### Differential rRNA presence

A maximum read length of 76 nt was used, so none of the rRNA types (smallest: 5S rRNA ≈120 nt and 5.8S rRNA ≈160 nt) were sequenced completely, and only rRNA-derived fragments smaller than 76 nt could be identified. When all data were collapsed to known complete rRNA sequences, DiP rRNAs showed patterns similar to those observed for miRNA, with a transient effect at T1 (*n* = 3), attenuation at intermediate time points, and a resurgent effect at T21 (*n* = 41). Most of these were specific 5S rRNAs (24/41, 59%) (Tables 2 and 4). Colostrum replacement appeared to affect 18S rRNA presence only, whereas 28S rRNA showed reduced serum presence at 21 days after milk replacement.

**Table 4.** Serum rRNAs showing differential presence in milk-replacer-fed goat kids compared with maternal-milk-fed goat kids.

	Fold change (log_2_)					Adjusted *P* value				
rRNA	T0	T1	T7	T14	T21	T0	T1	T7	T14	T21
5S rRNA-134	−0.17	−0.70	−0.40	0.27	**3.62**	0.96	0.59	0.79	0.94	**0.00**
5.8S rRNA-4	−1.94	−0.91	0.91	0.20	**3.48**	0.50	0.65	0.72	0.95	**0.00**
5S rRNA-314	−2.70	−0.61	1.69	1.53	**3.21**	0.58	0.80	0.49	0.79	**0.00**
5S rRNA-304	−0.08	0.01	0.33	0.66	**2.68**	0.96	0.99	0.79	0.79	**0.00**
5S rRNA-282	−0.82	0.38	1.39	−0.03	**2.66**	0.94	0.90	0.72	1.00	**0.02**
5S rRNA-335	−0.27	0.46	0.87	0.55	**2.57**	0.95	0.70	0.66	0.88	**0.00**
5S rRNA-105	−0.27	0.58	0.06	1.74	**2.42**	0.96	0.80	0.99	0.65	**0.00**
5.8S rRNA-2	−0.27	−0.01	0.94	0.86	**2.42**	0.95	0.99	0.49	0.59	**0.00**
5S rRNA-306	−0.15	0.51	0.39	0.67	**2.39**	0.96	0.65	0.76	0.87	**0.00**
5S rRNA-326	−1.06	−0.27	0.06	0.36	**2.23**	0.65	0.88	0.98	0.88	**0.00**
5S rRNA-300	−0.41	0.10	0.29	0.45	**2.17**	0.86	0.91	0.72	0.77	**0.00**
5S rRNA-164	−0.62	0.37	0.79	0.64	**2.12**	0.86	0.80	0.70	0.88	**0.00**
5S rRNA-100	−0.30	0.08	0.44	0.69	**2.06**	0.94	0.96	0.72	0.65	**0.00**
5S rRNA-98	−1.12	0.18	0.75	0.41	**2.01**	0.50	0.90	0.56	0.88	**0.00**
5S rRNA-40	0.17	−0.24	0.34	0.28	**1.74**	0.95	0.88	0.79	0.88	**0.00**
5S rRNA-119	−0.47	0.41	−0.21	0.21	**1.55**	0.86	0.69	0.93	0.94	**0.00**
5S rRNA-244	−0.46	0.21	−0.05	0.19	**1.50**	0.86	0.86	0.98	0.94	**0.00**
5S rRNA-239	−0.53	−0.06	0.06	0.16	**1.41**	0.86	0.96	0.98	0.94	**0.00**
5S rRNA-229	−0.63	−0.04	−0.12	0.36	**1.35**	0.86	0.96	0.97	0.88	**0.01**
5S rRNA-45	0.05	0.48	−0.10	0.30	**1.12**	0.96	0.56	0.97	0.88	**0.00**
18S rRNA-8	−0.06	**0.71**	0.21	0.16	**0.96**	0.96	**0.03**	0.72	0.88	**0.00**
18S rRNA-9	0.22	0.53	0.38	0.10	**0.94**	0.86	0.14	0.56	0.94	**0.00**
18S rRNA-4	−0.08	0.22	0.31	0.17	**0.80**	0.96	0.67	0.70	0.88	**0.00**
5S rRNA-264	0.02	−0.41	−0.08	0.20	**0.74**	0.97	0.55	0.97	0.88	**0.02**
18S rRNA-2	−0.06	0.39	0.19	0.16	**0.69**	0.96	0.33	0.72	0.88	**0.00**
18S rRNA-16	0.13	**0.67**	0.33	0.20	**0.61**	0.94	**0.01**	0.56	0.88	**0.00**
18S rRNA-12	0.15	**0.86**	0.24	0.05	**0.57**	0.95	**0.00**	0.72	0.95	**0.01**
18S rRNA-10	0.16	0.15	0.04	0.03	**−0.50**	0.90	0.80	0.98	0.98	**0.02**
28S rRNA-4	0.19	−0.34	−0.26	−0.57	**−0.72**	0.95	0.62	0.72	0.53	**0.02**
28S rRNA-2	0.04	0.29	0.08	0.09	**−1.06**	0.96	0.56	0.97	0.94	**0.00**
5.8S rRNA-8	0.09	−0.36	0.11	−0.12	**−1.33**	0.96	0.74	0.97	0.94	**0.00**
28S rRNA-3	0.37	−0.30	−0.12	−0.13	**−1.42**	0.88	0.83	0.97	0.94	**0.00**
5S rRNA-238	−0.66	0.23	−0.11	0.74	**−1.49**	0.86	0.90	0.98	0.73	**0.01**
5S rRNA-124	1.25	−0.75	−0.51	−1.69	**−1.51**	0.50	0.55	0.72	0.08	**0.01**
18S rRNA-13	0.22	−0.24	−0.39	−0.18	**−1.53**	0.95	0.90	0.79	0.94	**0.01**
18S rRNA-3	0.28	−0.14	−0.34	−0.44	**−1.55**	0.90	0.90	0.72	0.79	**0.00**
5S rRNA-245	−0.66	−0.36	0.42	0.04	**−1.61**	0.86	0.83	0.74	1.00	**0.00**
28S rRNA-1	0.45	−0.48	0.08	−0.39	**−1.62**	0.85	0.38	0.97	0.77	**0.00**
5S rRNA-190	−0.08	−0.05	−0.32	−0.50	**−1.64**	0.96	0.96	0.83	0.88	**0.00**
18S rRNA-5	−0.06	−0.06	0.00	−0.13	**−1.79**	0.97	0.96	1.00	0.95	**0.02**
5S rRNA-113	−0.44	−0.26	−0.62	−0.67	**−2.15**	0.95	0.90	0.72	0.88	**0.00**

Notes: Bold text, miRNAs that are differential at a given timepoint with *p* < 0.05.

**Figure 7.**
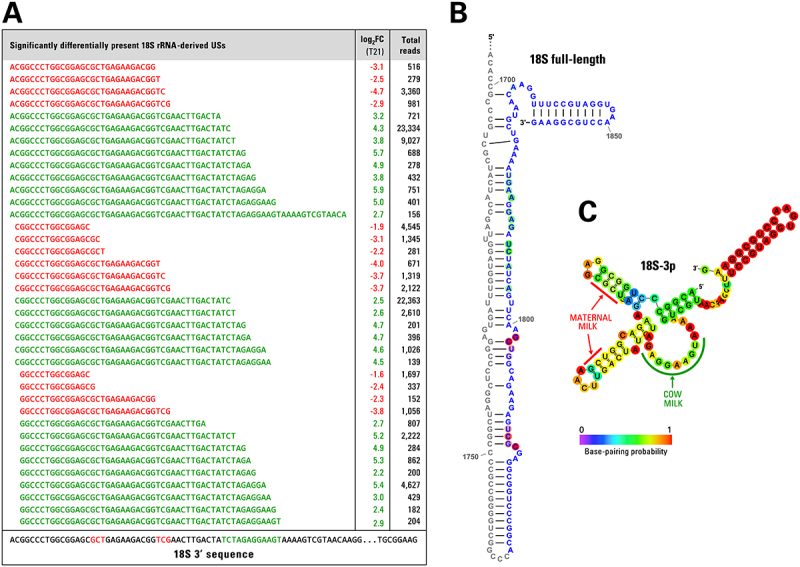
Differential presence and structure of 18S rRNA-3′ (18S-3p) – derived fragments. (A) Significantly differentially present unique sequences (USs) derived from the 18S-3p region, detected in serum from kids fed cow milk compared to those fed maternal (goat) milk (adjusted *p* < 0.05). Sequences are aligned against the full 18S-3p region (bottom). Log_2_ Fold change values (T21) and total counts across all samples are indicated. Red sequences were more abundant in maternal milk – fed animals, while green sequences were more abundant in cow milk – fed animals. (B) Depicts the 3’ terminal region (1,696–1,860) of the *Capra hircus* full-length 18S rRNA secondary structure, retrieved from RNAcentral (rnacentral.Org; accession URS0001BB4381_9925). The 3p fragment produced by cleavage between positions 1,731–1,734 is highlighted in blue. Nucleotides coloured red and green mark the terminal positions of the fragments displayed in panel A, with colour intensity reflecting the abundance of the corresponding reads (dark, high abundance; light, low abundance). (C) the minimum free energy (MFE) secondary structure of the 18S-3p fragment (1,733–1,860) was generated by folding this region independently with RNAfold. This structure supports interpretation of the observed cleavage patterns under a model in which the 18S-3p fragment is initially produced by cleavage at positions 1,731–1,734 and then refolds, enabling subsequent processing at additional cleavage sites: two stem-loops for maternal milk and a bulge for milk-replacer-fed goat kids (cow milk). Nucleotides are shaded according to base-pairing probability. Arrows denote candidate regions of preferential cleavage, with red indicating maternal milk-associated patterns and green indicating cow milk-associated patterns.

For 18S rRNA, the 3p part is processed to produce an 18S-3p-5p fragment by cutting at specific sites within the RNA molecule (Figure 7). Milk replacement may affect this process. Fragments originating from cutting in the first and second hairpin showed reduced numbers in serum, whereas fragments originating from cutting at the third loop showed increased numbers. Size distribution of sequenced rRNA-derived fragments differed after milk replacement, with changes observed not only in sncRNA abundance but also in their processing patterns.

### Differential tRNA presence

Complete tRNAs are not sequenceable with sRNA-seq, probably due to 3’ modifications, such as the presence of an amino acid. Hence, the vast majority of tRNA sRNA-seq reads represent tRNA-derived fragments, as reflected by an average read length of approximately 28 nucleotides. Noteworthy is the differential serum presence of tRNAs. In contrast to the virtual absence of differential tRNA serum presence during normal development (Table 1), 42% of all possible tRNAs showed altered presence following milk replacement (Table 2). For tRNAs, no transient effect was observed after colostrum replacement (T1), but after milk replacement (T21) there were differences in serum tRNA-derived fragment presence (Table 5).

**Table 5.** Serum tRNAs showing differential presence in milk-replacer-fed goat kids compared with maternal-milk-fed goat kids.

tRNA anticodon	Amino acid	DiP US*	Fold change (log_2_)					Adjusted P value				
			T0	T1	T7	T14	T21	T0	T1	T7	T14	T21
ACG	Arg	0	−0.3	0.9	0.3	0.4	**1.7**	0.96	0.66	0.94	0.72	**0.00**
CTC	Glu	15	−0.1	0.6	0.4	0.7	**1.1**	0.97	0.66	0.81	0.18	**0.00**
GCC	Gly	16	−0.2	0.7	0.7	0.9	**1.7**	0.96	0.66	0.80	0.18	**0.00**
TCC	Gly	3	−0.1	0.4	0.4	0.5	**1.3**	0.97	0.83	0.80	0.38	**0.00**
CCC	Gly	5	−0.2	0.6	0.4	0.9	**1.1**	0.96	0.66	0.80	0.14	**0.01**
CAG	Leu	1	0.1	0.4	0.5	0.7	**1.7**	0.97	0.87	0.91	0.46	**0.00**
CTT	Lys	2	0.1	0.1	−0.1	0.1	**1.2**	0.97	0.94	0.94	0.91	**0.00**
CAT	Met	0	0.3	0.6	0.0	0.0	**1.0**	0.93	0.66	1.00	0.97	**0.02**
GCT	Ser	2	−0.1	0.5	1.1	1.0	**1.4**	0.97	0.83	0.47	0.18	**0.00**
TCA	Stop	1	0.4	0.5	−0.2	0.0	**1.1**	0.84	0.66	0.94	0.94	**0.01**
AAC	Val	8	−0.1	0.2	0.0	0.3	**1.8**	0.97	0.94	0.99	0.72	**0.00**
CAC	Val	7	−0.1	−0.1	−0.1	0.1	**1.7**	0.96	0.94	0.94	0.91	**0.00**
AAT	Ile	0	**1.5**	0.0	0.1	−0.3	0.0	**0.03**	0.97	0.94	0.72	0.99
GTT	Asn	3	0.0	−0.1	0.1	−0.9	**−0.9**	1.00	0.94	0.94	0.13	**0.02**
TTG	Gln	3	**1.2**	−0.4	−0.1	−0.9	**−0.9**	**0.03**	0.83	0.96	0.13	**0.04**
TGG	Pro	15	0.1	0.1	−0.4	−0.2	**−1.5**	0.97	0.94	0.80	0.72	**0.00**
AGG	Pro	12	−0.1	0.4	−0.6	−0.5	**−2.2**	0.97	0.87	0.80	0.59	**0.00**
TGA	Ser	1	0.3	1.3	0.2	0.3	**−1.6**	0.96	0.66	0.94	0.73	**0.01**
TGA (Mito)	Ser (Mito)	2	−0.2	−0.7	−0.3	−0.2	**−1.0**	0.96	0.66	0.91	0.73	**0.02**
CGT	Thr	1	0.0	−0.1	−0.2	−0.2	**−1.1**	1.00	0.94	0.94	0.77	**0.02**
GTA	Tyr	2	0.7	−0.1	−0.1	−1.0	**−1.1**	0.67	0.94	0.94	0.13	**0.02**
NA (Mito)	–	–	0.9	−0.2	0.0	−1.0	**−1.7**	0.79	0.94	1.00	0.35	**0.01**

Notes: *, number of differentially present unique sequences (DiP US); Mito, mitochondrial tRNA; NA, not annotated.

tRNA-derived fragments derived from 22 anticodons were differently present in our sequencing data, with both up- and downregulation observed at moderate fold changes. Differential presence of tRNA-derived fragments was observed according to their related amino acid, with two Val and three Gly anticodon tRNAs showing higher presence, and two Pro and two Ser anticodon tRNAs showing lower presence after milk replacement. Also, nuclear ribonuclease P (RNase P) involved in tRNA processing is significantly lower present in serum after milk replacement at T14 and T21 (Supplementary Table ST5).

## DISCUSSION

In dairy goat farming, removing newborn goat kids at birth and raising them using bovine colostrum and milk replacer is a common practice to control lactogenic infections such as CL, CAE and paratuberculosis caused by MAP [41]. This study examined the effect of milk replacement on circulating sncRNAs in goat kid serum using a goat twin model, in which one twin-kid was fed maternal milk and the other twin-kid received bovine milk replacer. Results indicated that goat colostrum, goat milk, bovine colostrum and milk replacer contained an abundant diversity of sncRNAs. The highest abundance and complexity of sncRNAs was observed in goat colostrum and the lowest in milk replacer. As milk replacer is a reconstituted spray-dried product containing a mixture of dairy and plant-based nutrients and additional supplements, processing of dairy components likely accounts for low abundance of sncRNAs observed in this product. Similarly, bovine colostrum was stored at −20°C and underwent at least one freeze-thaw cycle, possible affecting sncRNA stability [42].

In neonatal goat kids, the serum sncRNA transcriptome showed relatively limited complexity, consistent with findings from human studies [43,44]. Similar to the transient presence of bovine IgG antibodies after colostrum replacement, most sncRNA types display early, transient differential serum presence. However, a more prominent differential sncRNA serum presence effect was observed after milk-replacement at day 21. These appear to represent two distinct periods influenced by colostrum and milk replacement, as can also be concluded from the different miRNAs with differential serum presence in each period. These changes might also effect the developing immune response, as many of serum tRNA halves that are often implicated with the immune system [45] showed differential presence primarily after 21 days of milk replacement. These findings are relevant to interspecies colostrum and/or milk replacement practices. Unlike the transient IgG presence provided by early colostrum replacement, the main serum sncRNA effects after milk-replacement occur later. The milk-replacer induced effects may persist longer, and could influence multiple tissues of developing goat kids.

The influence of milk replacement is highlighted by different cut sites involved in processing of 18S-3p rRNA-derived fragments in serum of goat kids from the two groups. These rRNA-derived fragments are non-random products generated via specific cleavage and processing events and are increasingly recognized for their regulatory roles in gene expression [46], immune modulation [47] and cellular adaptation. Processing of 18S rRNA is thought to depend on specific sequence and structural features and to be tissue- and species-specific [46]. However, in this study, these 18S-3p fragments were present in very low quantities in bovine milk. This suggests that observed differences were likely caused by altered processing of caprine 18S rRNA-3p fragments in goats due to milk replacement, rather than by bovine 18S rRNA-3p fragments processed differently in caprine versus bovine milk. Although the exact cause and consequence of this altered rRNA processing is yet unclear, the occurrence of this phenomenon suggests the potential presence of other serum sncRNA changes that might be less obvious but still biologically relevant.

Other sncRNAs that were differentially present in the transcriptome data after colostrum and milk replacement included miRNAs. miRNAs are stably packaged, primarily in extracellular vesicles that are secreted from several tissues, including the mammary gland [20,48]. After oral ingestion, these miRNAs circulate in the bloodstream for at least six hours and are distributed to tissues including liver, spleen and the brain [20]. Evidence is accumulating that miRNAs are absorbed in biologically meaningful amounts from nutritionally relevant doses of cow and pig milk and that they can affect gene expression in various cell types [20,48–50]. In goat maternal milk, miR-486-5p was highly abundant. According to miRbase, this miRNA is conserved between goat and cow, and miR-486 has been experimentally shown to regulate lactation in cows by targeting the PTEN pathway [51]. This pathway plays an essential role in mammary gland development by regulating associated gene expression, including AKT and mTor, thereby enhancing milk synthesis and secretion. Since goat serum samples were taken at the start of lactation, this explains the high levels of circulating miR-486-5p.

In goat kid serum, transcripts of three miRNAs were differentially present at all time points in the maternal-milk-fed group: miR-29a-3p, miR-2889 and miR-6923-3p. Chi-miR-29a is highly expressed in mammary glands of lactating goats [52,53]. Several studies have shown that this miRNA regulates cell proliferation and the immune response towards intracellular bacteria by targeting interferon-γ [52,54–56]. More specifically, miR-29a plays a role in immune regulation during infections with *Mycobacterium tuberculosis*, and has been proposed as diagnostic marker for rapid detection of tuberculosis patients [57]. This suggests that miR-29a may be directly transferred from milk produced in the mammary glands to goat kids, and that it is involved in immune regulation of intracellular mycobacterial infections.

The sequence of chi-miR-2889 is conserved in cow (bta-mir-2904). An epigenetic study showed that *Mycobacterium bovis* infection led to hypomethylation of specific regions in CD4+ T-cells. In general, hypomethylation leads to induced expression of RNAs. miR-2904 was one of the miRNAs located near hypomethylated regions, and therefore might play a regulatory role in T-cell functional responses during *Mycobacterium bovis* infection [58].

The third miRNA that was affected at all time points is miR-6923-3p. The sequence of this miRNA is conserved in humans (hsa-miR-1224) and has a variety of functions including regulation of lipid metabolism and negative regulation of TNF-α [59]. After *Mycobacterium bovis* BCG infection, this miRNA is induced in exosomes of human macrophages [60], suggesting that it plays a role in the host response after BCG infection. Hence, all three identified miRNAs that were differentially present at all time points have been linked to immune regulation of intracellular, mycobacterial infections. Besides, all three miRNAs were present in maternal milk, this could point to direct transfer from mother to offspring.

In the milk replacement group, two miRNAs were differentially present at all time points: chi-miR-6027-5p and chi-miR-6651-5p. The sequence of miR-6027-5p is similar to tomato sly-miR-6027, which plays a role in temperature adaptation and drought responses [61–63]. The sequence of miR-6651-5p is also found mainly in plants, but its function appears to be unknown. Since milk replacers are partially composed of plant-based nutrients, this presence of these miRNAs in serum of goat kids from the milk replacement group likely reflects this plant origin.

Comparison of sncRNAs in serum of goat kids fed with colostrum and milk replacers versus maternal milk showed that miRNA profiles were altered both at early and at later time points. Replacement consistently affected expression of miRNAs that target mRNAs in multiple pathways, including cell development, glucose metabolism and the differentiation and development of the nervous system. This is consistent with findings reported in humans [18].

Summarized, milk replacement altered miRNA profiles in serum of goat kids, potentially impacting development and immunity. Mother goats were vaccinated against MAP before conception, resulting in a broad adaptive immune response against this intracellular bacterium. It is possible that maternal MAP vaccination induced the presence of miRNA previously linked to immune responses against mycobacterial infections. These observations are in line with earlier observations regarding direct maternal transfer of antigen-specific maternal immunity to offspring. Results of the previously published experiment indicated a clear difference in adaptive immunity in kids from the maternal-fed group versus the bovine replacer-fed group regarding MAP specific cellular responses and antibody levels [7].

Hence, maternal transfer of miRNAs might contribute to immunity towards MAP, although other environmental factors could also modulate miRNA profiles, enhancing adaptation and resilience of kids to their specific environments [17,18,20,48,64,65]. Other developmental and metabolic pathways were also affected. Future studies should explore the biological implications of these findings in more detail.

It is important to note that sRNA-seq only detects sequenceable sncRNAs. Certain sncRNA, such as aminoacylated tRNAs, are poorly captured by RNA-seq, which hampers a comprehensive analysis of serum sncRNA. In addition, the relatively small sample size and lack of independent validation should be considered when interpreting the results. High sequence identity between bovine and caprine sncRNAs further prevented reliable discrimination of dietary and endogenous RNA sources using sequencing alone. Although many bovine and goat sncRNA sequences are identical, this does not imply functional equivalence, as species-specific RNA modifications may alter RNA structure and biological activity.

Future studies could focus on optimizing milk and colostrum management strategies to preserve the transfer of beneficial sncRNA from dam to offspring. These data may support the development of evidence-based rearing practices that maintain the benefits of bioactive RNAs while minimizing infectious disease transmission. This requires larger cohorts, independent validation and complementary analytical approaches, as well as integration of longitudinal health and performance data. Functional analyses of key miRNAs and tRNA- and rRNA-derived fragments will be important to determine how specific sncRNA signatures relate to immune competence, disease susceptibility (e.g. to neonatal MAP infection), growth, and metabolic programming in goat kids. This will allow a more accurate resolution of the origin, transfer, and functional relevance of milk-derived sncRNAs in neonatal development.

## Conclusions

This study indicates that maternal interspecies colostrum and milk replacement affects circulating sncRNAs in serum of neonatal goat kids. Goat colostrum, goat milk, bovine colostrum, and milk replacers all contained diverse sncRNAs, with processing and storage influencing their abundance and stability. In serum of neonatal goats, milk replacement altered profiles of rRNA-derived fragments and miRNAs, some of these are involved in immune regulation, metabolism, and development. Although high sequence similarity between bovine and goat sncRNAs prevented definitive conclusions about direct transfer of bovine milk sncRNAs to goat kids serum, the observed differences indicate that the origin and composition of milk may influence circulating sncRNAs. Several miRNAs present in maternal milk were consistently found in offspring serum, suggesting potential maternal transfer and a role in immune regulation, including responses to mycobacterial infections. Further studies are required to clarify the biological implications of these changes in relation to early immune function and developmental processes in neonatal goat kids.

## Supplementary Material

Supplemental Material

## Abbreviations

CC: cow colostrum group
DiP: differential presence
DiPR(s): differential presence of sncRNA(s)
GC: goat colostrum group
GO: Gene Ontology
KEGG: Kyoto Encyclopedia of Genes and Genomes
LncRNA: long non-coding RNA
MAP: Mycobacterium avium subspecies paratuberculosis
miRNA: microRNA
PCA: principal component analysis
PPD-A: purified protein derivative from Mycobacterium avium
rRNA: ribosomal RNA
sRNA-seq: small RNA sequencing;
sncRNA: small non-coding RNA
tRNA: transfer RNA
US(s): unique sequence(s)
