# Supplementary material for: Impact of interspecies colostrum and milk replacement on circulating sncRNA dynamics of neonatal goat kids
Source: RNA Biol. 2026 Jun 26;23(1):1–21. doi: 10.1080/15476286.2026.2692293 (PMC13313187; doi:10.1080/15476286.2026.2692293)
Supplement: Supplemental Material [file KRNB_A_2692293_SM4394.zip › Supplemental Figures.pdf]

A

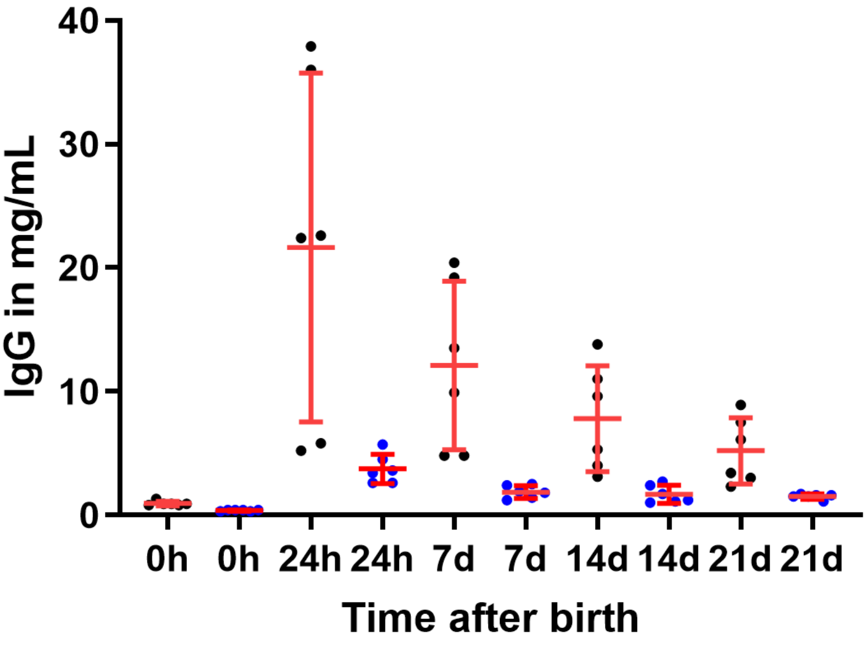

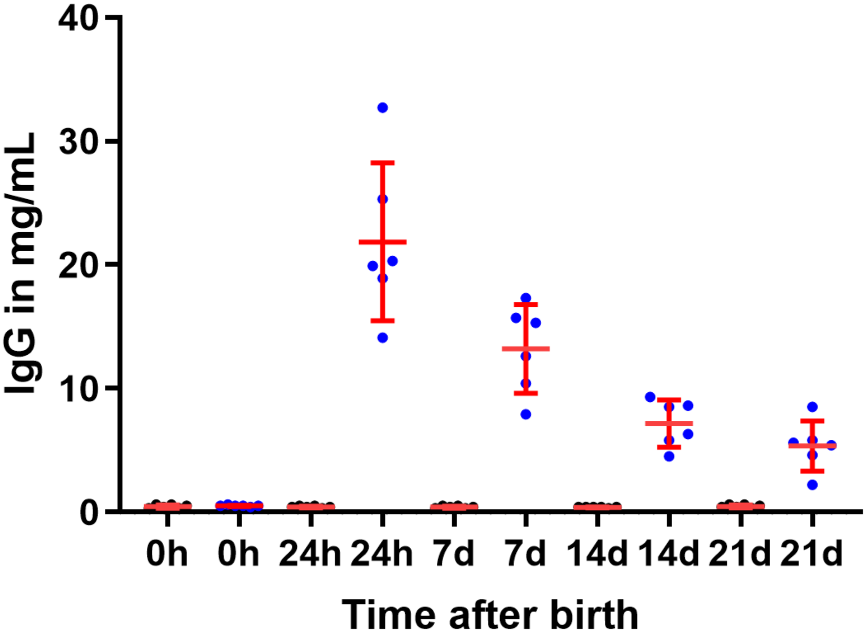

Supplemental Figure SF1. The graphs show the serum concentration of caprine (black dots) and bovine (blue dots) IgG expressed as mg/ml serum. Panel A shows the IgG concentration in serum from the goat kids that were fed maternal colostrum (GC group). Panel B shows the IgG concentration in serum from the goat kids that were fed cow colostrum (CC group). Both caprine (black dots) and bovine (blue dots) IgG was measured in all serum samples from the indicated time points following birth of the kids.

A

B

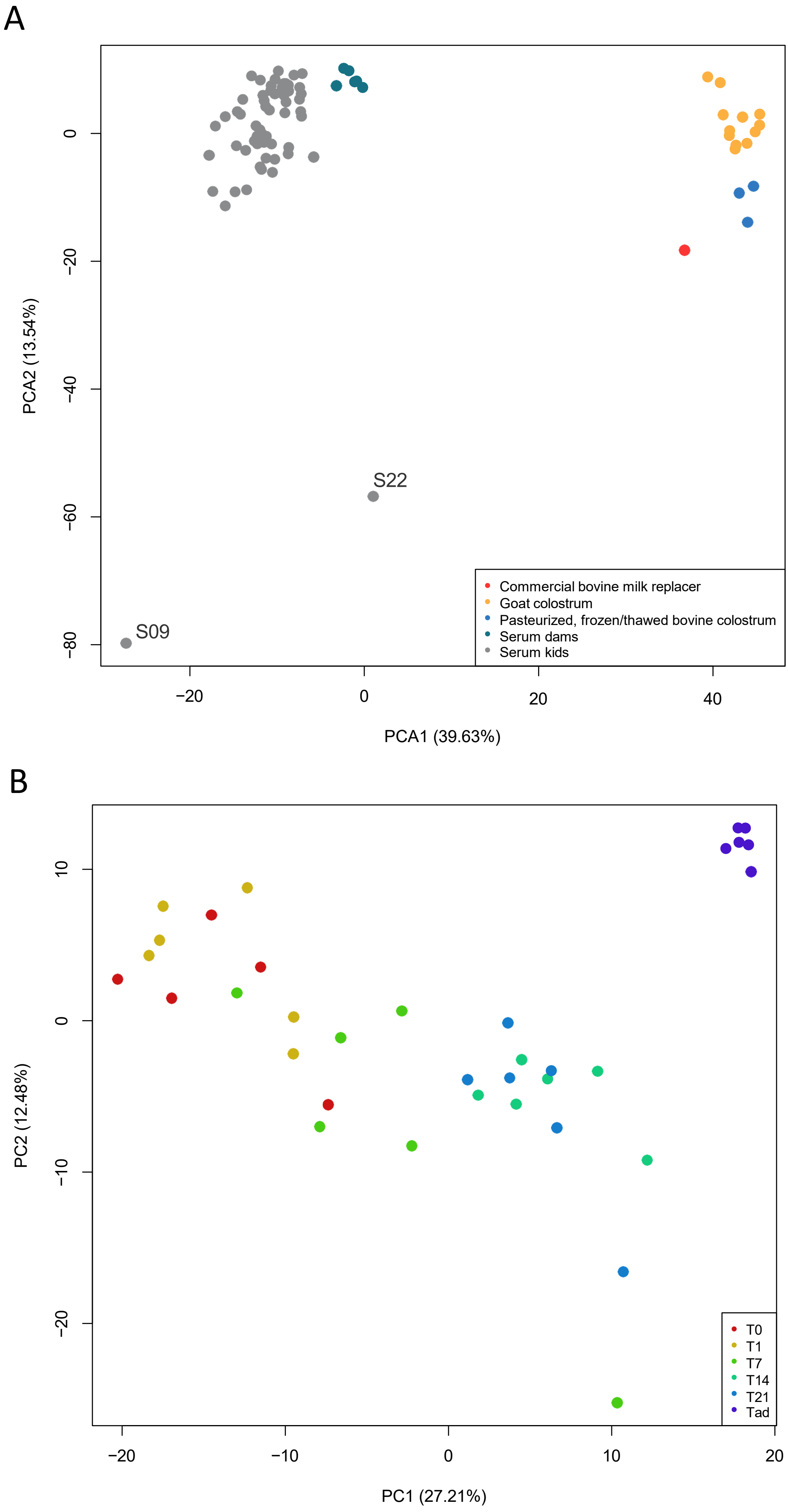
A
S22
S09
PCA2 (13.54%)
PCA1 (39.63%)
0
−20
−40
−60
−80
−20
0
20
40
Commercial bovine milk replacer
Goat colostrum
Pasteurized, frozen/thawed bovine colostrum
Serum dams
Serum kids
B
PC2 (12.48%)
PC1 (27.21%)
10
0
−10
−20
−20
−10
0
10
20
T0
T1
T7
T14
T21
Tad

Supplemental Figure SF2. Data Quality check - Principal Component Analysis (PCA) analysis of miRNA presence. A: One of the sRNA-seq data quality checks, a Principal Component Analysis (PCA) of all samples in the experiment based on the preprocessed read counts of all miRNAs present in any of the samples in the experiment, revealed two outlier samples: sample 9 (serum of kid from dam 93011 @ T0) and sample 22 (serum of kid on bovine milk replacement from dam 93011 @ T1) that clearly did not cluster with the other kid serum samples. In sample 22 this was caused by an insufficient sncRNA yield from the serum sample, but for sample 9 no obvious cause could be determined. Due to the extreme aberrant variation of the miRNA presence in these samples, they were omitted from any further analysis of this experiment. B: PCA of the time series serum samples from developing kids (T0 – T21) that were fed maternal goat milk plus the serum samples of the mothers (Tad).

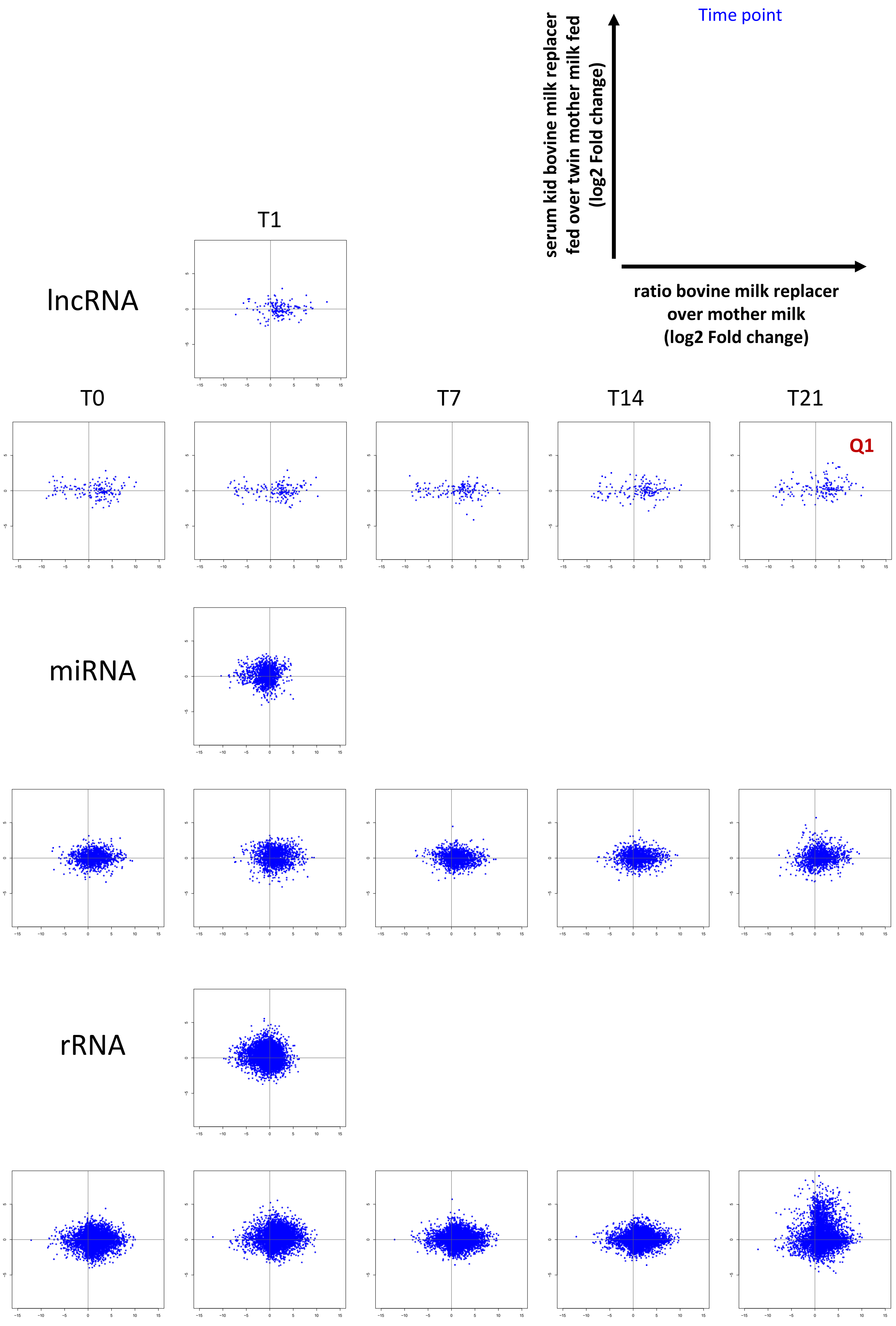
Time point
serum kid bovine milk replacer fed over twin mother milk fed (log2 Fold change)
ratio bovine milk replacer over mother milk (log2 Fold change)
T1
lncRNA
T0
T7
T14
T21
Q1
miRNA
rRNA

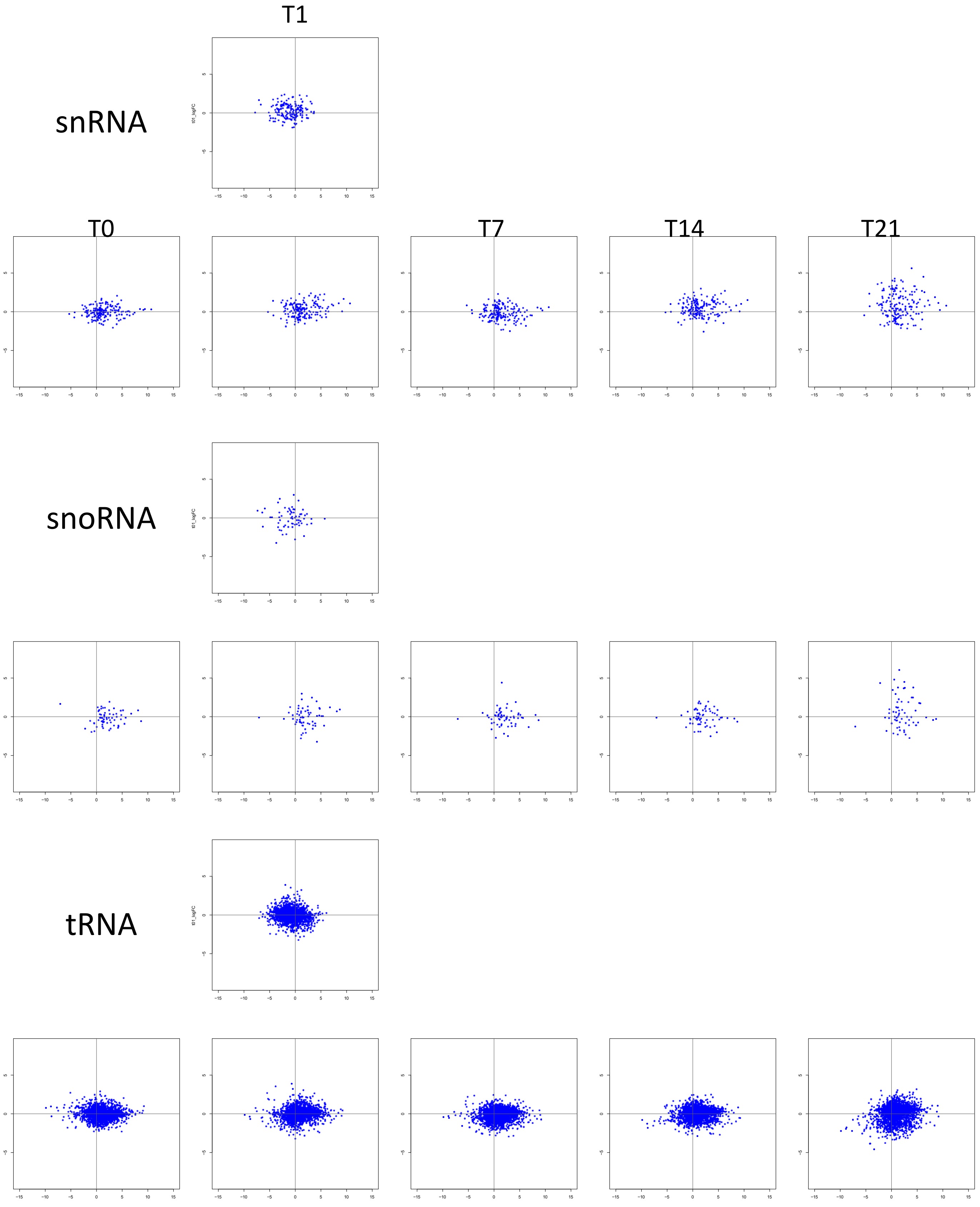

Supplemental Figure SF3. Relation between milk presence and serum presence of ncRNAs. Scatterplots to illustrate the relation between ncRNA milk presence and serum presence. Examples of ncRNA USs presence (i.e. read counts) in a scatterplots per time point based on the fold change (log2) ratio of bovine milk replacer over mother milk (Tgm Figure 1) (x-axis) and the fold change (log2) ratio of serum kid bovine milk replacer fed over twin mother milk fed (y-axis). In the second column, the upper scatterplot is calculated with the fold change (log2) ratio of bovine colostrum over the fold change (log2) ratio of goat colostrum (Tgc Figure 1). The numbers of Q1 are summarized in Figure 3.

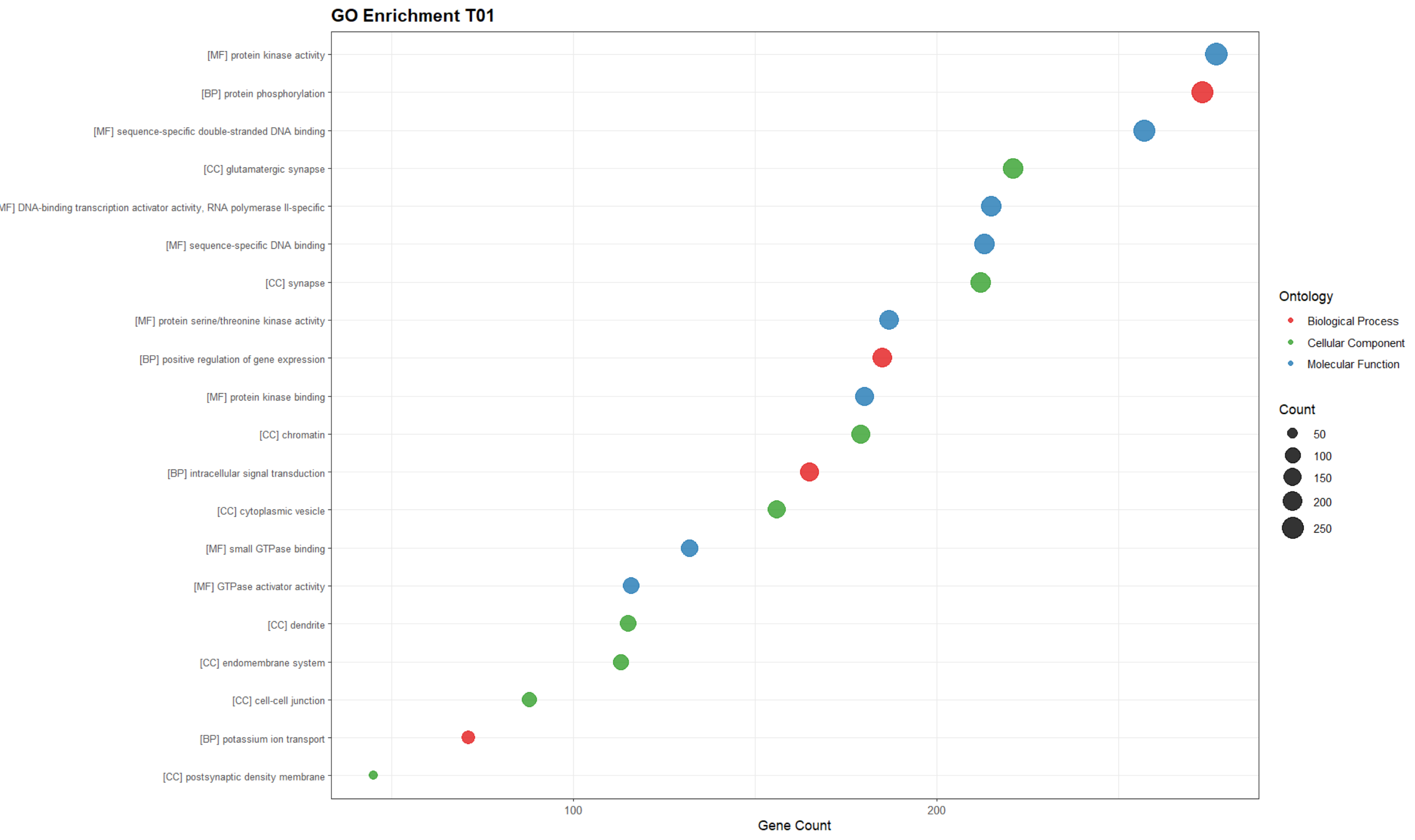

GO Enrichment T01
[MF] protein kinase activity
[BP] protein phosphorylation
[MF] sequence-specific double-stranded DNA binding
[CC] glutamatergic synapse
MF] DNA-binding transcription activator activity, RNA polymerase II-specific
[MF] sequence-specific DNA binding
[CC] synapse
[MF] protein serine/threonine kinase activity
[BP] positive regulation of gene expression
[MF] protein kinase binding
[CC] chromatin
[BP] intracellular signal transduction
[CC] cytoplasmic vesicle
[MF] small GTPase binding
[MF] GTPase activator activity
[CC] dendrite
[CC] endomembrane system
[CC] cell-cell junction
[BP] potassium ion transport
[CC] postsynaptic density membrane
100
200
Gene Count
Ontology
Biological Process
Cellular Component
Molecular Function
Count
50
100
150
200
250

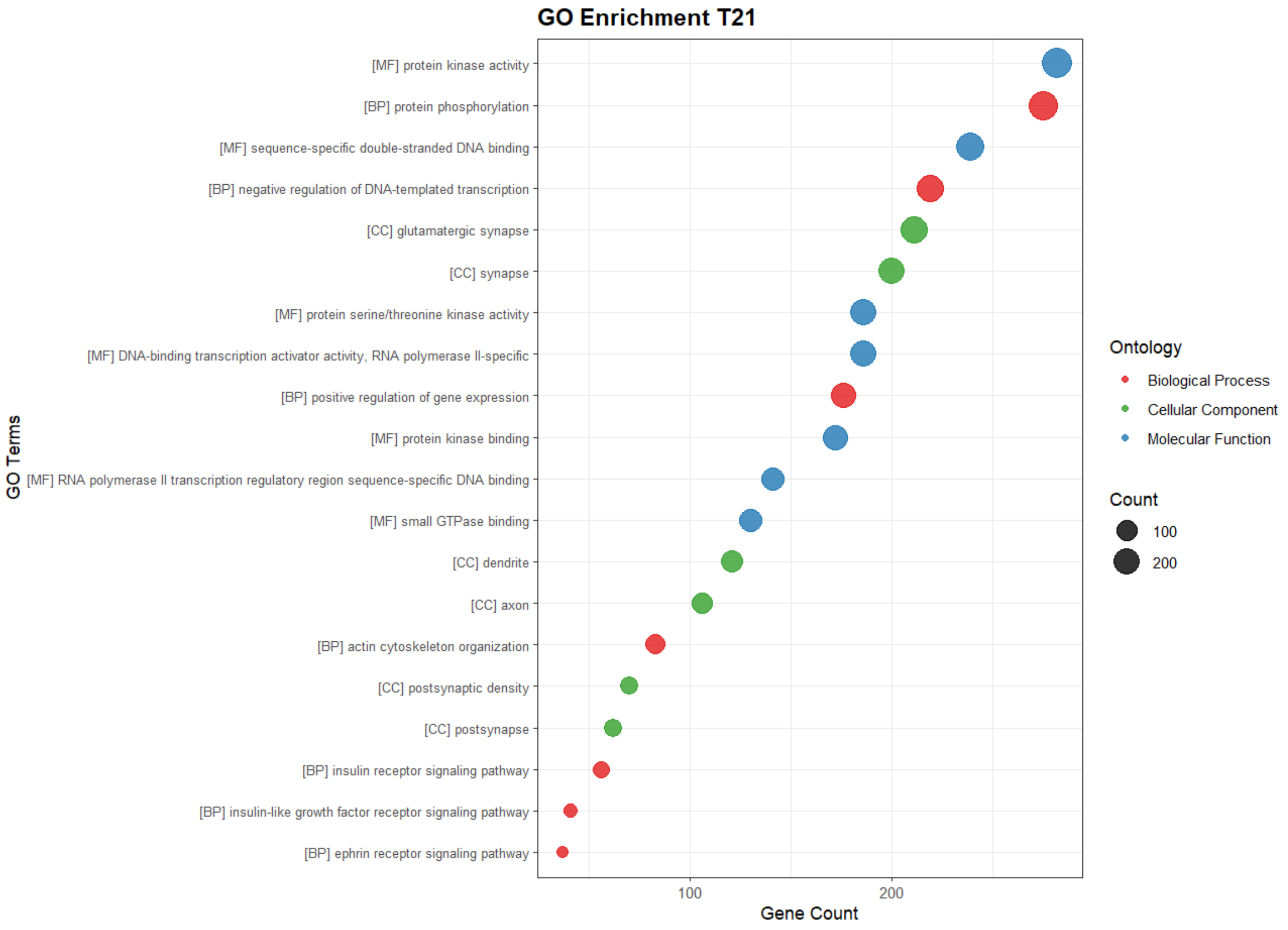

GO Enrichment T21
GO Terms
[MF] protein kinase activity
[BP] protein phosphorylation
[MF] sequence-specific double-stranded DNA binding
[BP] negative regulation of DNA-templated transcription
[CC] glutamatergic synapse
[CC] synapse
[MF] protein serine/threonine kinase activity
[MF] DNA-binding transcription activator activity, RNA polymerase II-specific
[BP] positive regulation of gene expression
[MF] protein kinase binding
[MF] RNA polymerase II transcription regulatory region sequence-specific DNA binding
[MF] small GTPase binding
[CC] dendrite
[CC] axon
[BP] actin cytoskeleton organization
[CC] postsynaptic density
[CC] postsynapse
[BP] insulin receptor signaling pathway
[BP] insulin-like growth factor receptor signaling pathway
[BP] ephrin receptor signaling pathway
100
200
Gene Count
Ontology
Biological Process
Cellular Component
Molecular Function
Count
100
200

Supplemental Figure SF4. Gene Ontology overrepresentation analysis of DiPRs between maternally fed and cow-fed goat kids at T01 and T21. The top 20 GO terms that are overrepresented are shown, including Biological Process (BP), Molecular Function (MF), and Cellular Component (CC) categories at T01 and T21.
