# Supplementary material for: Impact of interspecies colostrum and milk replacement on circulating sncRNA dynamics of neonatal goat kids
Source: RNA Biol. 2026 Jun 26;23(1):1–21. doi: 10.1080/15476286.2026.2692293 (PMC13313187; doi:10.1080/15476286.2026.2692293)
Supplement: Supplemental Material [file KRNB_A_2692293_SM4394.zip › Supplemental Materials and Methods.docx]

# Supplemental Materials and Methods

***Ethical approval for animal experiment***

The ethical approval was originally obtained for a study in investigating the transfer of functional cellular immunity in the twin goat model. The results of this studied have been published in Robbers et al. 2022.

Under the regulations for the use of animals in scientific experiments, the previous study was conducted in accordance with the 3R (reduce, refine, replace) principles. This means that, whenever possible, it is stimulated to biobank materials from animal experiments for future studies i.e., in case new technologies become available and new hypothesis can be tested. The senior author (A.P. Koets) from the previous study in Robbers et al (2022) therefore biobanked the materials from that study.

The design of the study and the nature of the materials in the biobank enabled us to investigate the effects of small RNA in the twin goat model in the current study, without repeating the original animal study. In this way we contribute to the 3R (reduce) principles. As a consequence, we report the license number from the earlier study to indicate that the samples originate from biobanked material from a licensed animal experiment.

### Reference:

Robbers, L., R. van de Mheen, L. Benedictus, R. Jorritsma, M. Nielen, H.J.C. Bijkerk, S.G. van der Grein, L. Ravesloot, and A.P. Koets. 2022. Evidence for transfer of maternal antigen specific cellular immunity against Mycobacterium avium ssp. paratuberculosis via colostrum in a goat twin model. Vet Immunol Immunopathol 246:110402. https://doi.org/10.1016/j.vetimm.2022.110402.
